# Isolated Cricoid Fracture After Intubation

**DOI:** 10.7759/cureus.74811

**Published:** 2024-11-30

**Authors:** Cláudia Pereira, Rafaela Lopes Freitas, Cristina Pereira, Nuno Oliveira, Daniela Carvalho

**Affiliations:** 1 Anesthesiology, Hospital Pedro Hispano, Matosinhos, PRT; 2 Internal Medicine, Hospital Pedro Hispano, Matosinhos, PRT; 3 Otolaryngology, Hospital Pedro Hispano, Matosinhos, PRT; 4 Intensive Medicine, Hospital Pedro Hispano, Matosinhos, PRT

**Keywords:** airway complications, computed tomography (ct ), cricoid abscess, isolated cricoid fracture, stridor, voice changes

## Abstract

Isolated cricoid fractures are exceedingly rare but can be life-threatening. Injuries caused by minor neck trauma related to external laryngeal manipulation or an inappropriate tube cuff size have been reported in the literature. Symptoms typically appear immediately after the traumatic episode. Voice changes, respiratory distress, or deglutition problems may be present, although some patients may initially be asymptomatic. Here, we report a case of a 28-year-old woman with a history of recurrent dysphonia and respiratory distress after an episode of voluntary drug intoxication requiring orotracheal intubation and mechanical ventilation. The initial diagnostic workup was apparently normal, but conservative treatment did not resolve the symptoms. The progressive stridor episodes ultimately led to the correct diagnosis of a posterior cricoid fracture and abscess. The airway was secure with a transient tracheostomy, and the fracture was managed conservatively with no sequelae. A high level of suspicion is essential for the timely identification of these injuries to minimize the associated morbimortality, particularly in situations without obvious neck trauma and insidious symptoms.

## Introduction

Laryngeal injuries are uncommon, with a reported incidence of one in 5000 to 137,000 emergency room visits. These are generally caused by significant trauma to the neck, often associated with intracranial injuries, penetrating neck injuries, cervical spine, and facial fractures. Isolated fracture of the cricoid cartilage is exceedingly rare; nonetheless, it can lead to life-threatening airway obstruction [1-3].

Patients may present with a range of symptoms, such as dyspnea, dysphonia, neck pain, dysphagia, odynophagia, and hemoptysis, typically developing immediately after the traumatic episode. However, some patients are asymptomatic at presentation, with up to one-third of patients presenting with no signs or symptoms in the initial 24-48 hours, which may cause a delay in diagnosis [1,2,4].

Morbidity from traumatic laryngeal injuries is associated with difficulty in maintaining airway patency and complications related to aspiration and phonation in up to 37% of patients [3,5].

The rare nature of this injury and lack of physician awareness contribute to the underdiagnosis of these fractures. We report the delayed diagnosis of an isolated cricoid fracture presenting with intermittent symptoms and without obvious neck trauma. Our case highlights the necessity of prompt identification and management of laryngeal fractures to avoid the morbidity and mortality associated with this injury and improve patient outcomes.

## Case presentation

A 28-year-old woman was admitted to our emergency department with a decreased state of consciousness due to drug intoxication. She had a previous history of voluntary intoxication, anorexia nervosa, anemia, and bipolar disorder. On arrival, she presented with features of a sedative-hypnotic syndrome with a Glasgow Coma Score (GCS) of 8/15 and poor reactive mydriatic pupils. Her vitals were otherwise stable. No signs of ecchymosis, fractures, or emphysema were noted on the physical exam. Laboratory tests showed evidence of benzodiazepines in the urine, with no other relevant alterations. After primary examination, to maintain the airway, she was intubated under intravenous anesthesia and muscle relaxation with a 7 mm diameter endotracheal tube by an anesthetist, with no registered complications.

The intensive care unit (ICU) and toxicology center were consulted, and the appropriate treatment was administered. She was then transferred to the ICU, where she remained for three days in mechanical ventilation, with only some periods of agitation and no other occurrences. After extubation, she developed mild dysphonia and odynophagia that improved with a corticosteroid regimen. She was transferred to the medical ward, but immediate follow-up was not possible because the patient was discharged against clinical advice.

Later, dysphonia worsened, and signs of respiratory difficulty led to an evaluation in another hospital, where she was again prescribed oral corticosteroids.

Two weeks after the initial admission, she returned to our hospital with symptoms of shortness of breath and dysphonia. The Ear, Nose, Throat (ENT) doctor evaluation included a flexible fiberoptic nasopharyngoscopy, which revealed mild vocal cord dysfunction with a preserved glottic lumen and intact mucosa throughout the pharynx and larynx.

Symptoms persisted despite conservative treatment. She was reassessed by ENT doctors, and fiberoptic evaluation was similar. Due to the significant subjective complaints of the patient, a non-contrast computed tomography (CT) scan of the neck was performed. No apparent relevant findings were noted, and she was discharged with oral steroids and nebulized bronchodilators.

Three days later, she sought help due to progressively worsening dysphonia, dyspnea, and stridor. The patient was re-admitted to the ICU for surveillance and further investigation. On the third admission day, urgent intubation was necessary during an episode of intense stridor. Laryngoscopy was performed with the C-Mac video laryngoscope; evident glottic narrowing was visualized during intubation, limiting the endotracheal tube size to 6mm in diameter. On the following day, she was taken to the operating room. Intraoperative direct laryngoscopy demonstrated purulent secretions in the larynx, marked edema of the right arytenoid and aryepiglottic fold, and edema of the vocal cords and subglottic region leading to a considerable reduction in the airway caliber. A temporary tracheostomy between the second and third tracheal rings was performed to maintain airway patency.

**Figure 1:**
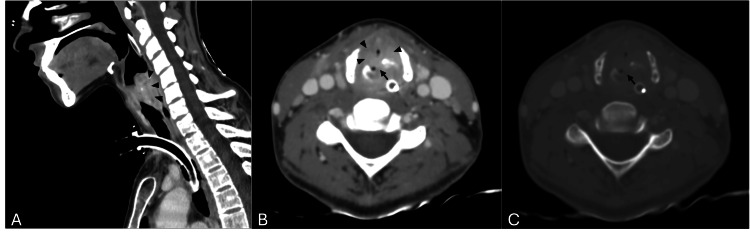
Neck CT after tracheostomy (A) Sagittal and (B, C) axial computed tomography images demonstrating a displaced, vertically oriented complete fracture of the cricoid cartilage (arrow), associated with a subglottic abscess (arrowheads).

A new CT scan was taken and demonstrated a complete axial fracture of the cricoid cartilage with a 6mm deviation and significant airway narrowing due to a horseshoe-shaped subglottic abscess (Figure 1). After carefully reviewing the previous images, we agreed that these changes were already present, although more discreet.

The patient's airway symptoms significantly improved following the procedure, allowing the transition to spontaneous ventilation through the tracheostomy in the hours following the postoperative period.

The case was discussed with the ENT team. Conservative medical treatment was chosen, starting with antibiotic treatment. However, the possibility of surgery to drain the abscess or reduce the fracture was not ruled out.

She was transferred to the otolaryngology department for continuation of treatment. Progress was monitored through repeated fiberoptic laryngeal examination and reassessment CT scans. During this period, she was initially fed through a nasogastric tube and started sessions of speech therapy to help improve vocalization and deglutition.

Sixteen days after the tracheostomy, a normal glottic lumen was visible, with practically normal vocal cord mobility. The patient was then discharged home asymptomatic, with a closed tracheostomy and a course of oral antibiotics.

**Figure 2:**
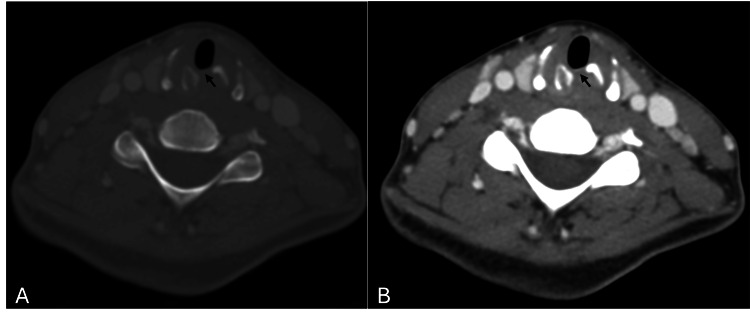
Neck CT two months after discharge (A, B) Axial computed tomography images showing complete resolution of the subglottic collection, and the known displaced fracture of the cricoid with sclerosis of the cricoid cartilage on the left (arrow).

At one-month reevaluation, the patient reported further improvement in symptoms, and fiberoptic laryngoscopy demonstrated only mild limitation in vocal fold abduction. On the follow-up visit two months after discharge, she was completely asymptomatic with normal vocal cord mobility and a complete resolution of the subglottic collection on the CT scan (Figure 2). 

## Discussion

Laryngeal injuries are uncommon as the mandible, sternum, and sternocleidomastoid muscle protect the cartilages [2]. Cricoid cartilage fracture is generally associated with other laryngeal fractures, most resulting from significant trauma, such as blunt neck trauma, strangulation, and suicidal hangings [6-11].

Isolated cricoid fractures are exceedingly rare but can be life-threatening. There are few reports of isolated fractures caused by minor neck trauma in the literature. Rare cases relate isolated cricoid fractures to intubation after external laryngeal manipulation or due to inappropriate cuffed tracheal tube size [12,13]. Non-traumatic laryngeal fractures are an exceptionally rare presentation, with only one case documenting spontaneous cricoid cartilage fracture reported in the literature to this date [14].

These fractures pose a diagnostic challenge due to the regular appearance of the neck and subtle associated symptoms. The cricoid cartilage is essential for phonation and airway patency. The phonatory function is dependent on the posterior part of the cricoid, where the arytenoids are located, and the airway is maintained by the anterior part [15]. The presentation may be in the form of voice changes, severe stridor, and respiratory distress, which may follow immediately after the traumatic episode. Also, a considerable proportion of patients can be completely asymptomatic at first. Nonetheless, they can cause significant morbidity and sequelae associated with difficulty maintaining airway patency, phonation, and complications related to aspiration. On the physical examination, cervical swelling, ecchymosis, laryngeal tenderness, loss of anatomic landmarks, and subcutaneous emphysema must raise suspicion of laryngeal injury [1-5].

Primary investigations in the diagnosis of this condition in a stable and cooperative patient should include an endoscopic examination. Bedside flexible fiberoptic laryngoscopy is a simple and feasible option for the assessment of the airway status, glottic and laryngeal anatomy, vocal cord function, mucosal changes, and identification of displaced laryngeal structures. A CT scan of the neck is the preferred imaging modality for initial evaluation, allowing a rapid and accurate detection of laryngeal injuries. However, radiologic diagnosis of these fractures can be challenging due to anatomic variations or asymmetric ossification patterns. Examining both bone and soft tissue windows is essential. Fractures can be challenging to detect when there is minimal calcification of the cricoid cartilage, as may occur with young patients [16,17].

Our patient's clinical symptoms had an insidious course. The transitory voice changes and odynophagia were initially attributed to an acute laryngeal injury, given the context of mechanical ventilation and recent extubation. However, the correct follow-up of this hypothesis was not possible due to the anticipated hospital discharge. The cause of the recurrent dysphonia and respiratory distress was not evident at first. The fracture of the cricoid cartilage was missed on the initial CT scan. The unusual nature of this injury, coupled with the presence of subtle radiological changes in the absence of an obvious neck trauma, may have contributed to the missed diagnosis.

Radiologists must be familiar with laryngeal fracture patterns and related soft tissue abnormalities to ensure timely diagnosis of these injuries. In a retrospective review of patients with laryngeal fractures who underwent CT imaging [5], Buch et al. reported that cricoid fractures always occurred with soft tissue abnormalities. Our case is consistent with this observation. The correct diagnosis of the posterior cricoid fracture has become evident due to the formation of a subglottic collection. A cricoid abscess is the rarest form of laryngeal abscess. The pathologic mechanism leading to abscess formation is poorly understood but seems to be related to an immunocompromised state and mechanical trauma such as intubation and nasogastric tube placement [18], all of which were present in this patient’s case.

Thorough assessment and management of laryngeal injuries are crucial due to the risk of fatal outcomes and chronic sequelae. Treatment priorities are airway protection and maintenance of ventilation, though the optimal approach remains a topic of debate [1,2,17]. Isolated cricoid fractures can be monitored if the endolarynx remains intact and the airway is patent. Open reduction and fixation are recommended for displaced fractures [11]. In this case, the airway was secured with a transient tracheostomy. The ENT team opted for conservative management of the cricoid fracture and abscess.

The cause of the isolated cricoid fracture is unclear. A direct causal relationship with the intubation technique seems unlikely, as an airway-trained professional performed it with no external laryngeal manipulation. However, it cannot be ruled out. We hypothesize that the periods of agitation during the three-day stay in the ICU while being intubated with both orotracheal and nasogastric tubes, could have been the source of mild trauma causing the fracture. It has been shown that endolaryngeal trauma predisposes to scarring and deformation of the cricoid cartilage [19]. Additionally, the patient’s anorexia could be associated with bone fragility and increased risk of fracture [20]. The mucosal and cartilaginous injury resulting from this fracture led to the formation of a collection, clinically manifesting with progressive dyspnea and stridor.

## Conclusions

Isolated cricoid fractures are exceedingly rare and can be life-threatening since the cricoid cartilage is essential for airway stability and integrity. This case demonstrates several pitfalls in the diagnosis of an isolated cricoid injury. It can be delayed or missed in situations without obvious neck trauma associated with an insidious clinical course and intermittent symptoms. A high level of suspicion is required to minimize the associated morbimortality.
